# Use of an invertebrate animal model (*Aplysia californica*) to develop novel neural interfaces for neuromodulation

**DOI:** 10.3389/fnins.2022.1080027

**Published:** 2022-12-22

**Authors:** Junqi Zhuo, Jeffrey P. Gill, E. Duco Jansen, Michael W. Jenkins, Hillel J. Chiel

**Affiliations:** ^1^Department of Biomedical Engineering, Case Western Reserve University, Cleveland, OH, United States; ^2^Department of Biology, Case Western Reserve University, Cleveland, OH, United States; ^3^Department of Biomedical Engineering, Vanderbilt University, Nashville, TN, United States; ^4^Biophotonics Center, Vanderbilt University, Nashville, TN, United States; ^5^Department of Neurological Surgery, Vanderbilt University, Nashville, TN, United States; ^6^Department of Pediatrics, Case Western Reserve University, Cleveland, OH, United States; ^7^Department of Neurosciences, Case Western Reserve University, Cleveland, OH, United States

**Keywords:** *Aplysia*, thermal inhibition, infrared neural modulation, small-diameter axon block, infrared neural inhibition, infrared neural stimulation, neuromodulation

## Abstract

New tools for monitoring and manipulating neural activity have been developed with steadily improving functionality, specificity, and reliability, which are critical both for mapping neural circuits and treating neurological diseases. This review focuses on the use of an invertebrate animal, the marine mollusk *Aplysia californica*, in the development of novel neurotechniques. We review the basic physiological properties of *Aplysia* neurons and discuss the specific aspects that make it advantageous for developing novel neural interfaces: First, *Aplysia* nerves consist only of unmyelinated axons with various diameters, providing a particularly useful model of the unmyelinated C fibers in vertebrates that are known to carry important sensory information, including those that signal pain. Second, *Aplysia’s* neural tissues can last for a long period in an *ex vivo* experimental setup. This allows comprehensive tests such as the exploration of parameter space on the same nerve to avoid variability between animals and minimize animal use. Third, nerves in large *Aplysia* can be many centimeters in length, making it possible to easily discriminate axons with different diameters based on their conduction velocities. *Aplysia* nerves are a particularly good approximation of the unmyelinated C fibers, which are hard to stimulate, record, and differentiate from other nerve fibers in vertebrate animal models using epineural electrodes. Fourth, neurons in *Aplysia* are large, uniquely identifiable, and electrically compact. For decades, researchers have used *Aplysia* for the development of many novel neurotechnologies. Examples include high-frequency alternating current (HFAC), focused ultrasound (FUS), optical neural stimulation, recording, and inhibition, microelectrode arrays, diamond electrodes, carbon fiber microelectrodes, microscopic magnetic stimulation and magnetic resonance electrical impedance tomography (MREIT). We also review a specific example that illustrates the power of *Aplysia* for accelerating technology development: selective infrared neural inhibition of small-diameter unmyelinated axons, which may lead to a translationally useful treatment in the future. Generally, *Aplysia* is suitable for testing modalities whose mechanism involves basic biophysics that is likely to be similar across species. As a tractable experimental system, *Aplysia californica* can help the rapid development of novel neuromodulation technologies.

## Introduction

New tools for monitoring and manipulating neural activity will be critical for understanding the function of neural circuits and for treating nervous system diseases. In recent years, many new approaches have been developed whose functionality, specificity, and reliability have steadily improved. Here we review the use and utility of an invertebrate animal, the marine mollusk *Aplysia californica*, to develop novel neural interface technologies for recording and/or modulating neural activity. After briefly describing the advantages of *Aplysia’s* physiology for developing novel neural interfaces, we will focus on a specific example that illustrates the power of *Aplysia* for accelerating technology development: using heat to selectively inhibit small-diameter unmyelinated axons.

In the 1960s, *Aplysia’s* large, pigmented neurons attracted the attention of neuroscientists who wanted to better understand the biophysical properties of individual neurons. Early studies demonstrated that the neurons could be repeatedly and reliably identified by their location and anatomy, their electrophysiological properties, their synaptic inputs and followers, their biochemical properties, and their functional roles as sensory neurons, motor neurons, and interneurons (Frazier et al., 1967; Kandel, 1976). The neurons’ somata (cell bodies) are very large (about 30 to 500 μm in diameter). A major advantage of *Aplysia’s* neurons is that its soma is electrically compact just like vertebrate neurons (Shapiro et al., 1980; Connor et al., 1986), allowing the neurites to be electrically manipulated from a single control point. In contrast, arthropod neurons (e.g., crustacean and insect neurons) are often not electrically compact and their somata are not often excitable (Smarandache-Wellmann, 2016). Thus, in *Aplysia*, researchers can easily monitor the neuronal activity *via* the soma, which is the largest and most accessible part of the cell. Furthermore, because the *Aplysia* neuron’s soma is electrically excitable, activating or inhibiting neurons at these locations can turn the rest of the neuron on or off, making it possible to test the ability of novel neural interfaces to monitor and manipulate individual identified neurons in a neural circuit. The neurons are arranged in groups (referred to as ganglia) that generally contain ∼2,000 neurons. The entire nervous system contains approximately 20,000 neurons. Studies of neural circuitry and behavior in *Aplysia* have clarified the neural, biophysical, and molecular basis of learning and memory (Mayford et al., 2012; Tam et al., 2020), sleep (Keene and Duboue, 2018; Thiede et al., 2021), and complex behaviors such as mating (Painter et al., 1991) and feeding (Kandel, 1979; Susswein and Chiel, 2012). Many novel technologies can take advantage of the deep understanding of neural circuitry controlling behavior in *Aplysia* to study the effects of manipulating single neurons or small numbers of neurons on behavior in both reduced preparations and in intact, behaving animals.

## Advantages and examples of using *Aplysia* for developing novel neural interfaces

In addition to the advantages that led researchers to initially use the *Aplysia*’s nervous system in their work, there are several other reasons to use *Aplysia*. Here, we highlight the rationale for using *Aplysia* for the development of novel interface technologies that focus on monitoring or manipulating axons.

First, *Aplysia* does not produce myelin. Nerves connecting the different ganglia to one another and to the animal’s body consist of populations of unmyelinated axons whose diameters range from less than 1 μm to over 10 μm. Thus, one can examine the effects of a variety of techniques for manipulating axonal activity in a pure population of unmyelinated axons that vary greatly in diameter. This makes the animal a particularly useful model of the unmyelinated C fibers in vertebrates that are known to carry important sensory information, including those that signal pain. For example, *Aplysia* nerves helped researchers explore the feasibility of selective neural inhibition using high-frequency alternating current (HFAC), which has been studied for decades for its capability to reversibly and repeatedly block neural conduction in frogs, rats, and cats (Kilgore and Bhadra, 2004; Bhadra and Kilgore, 2005; Bhadra et al., 2006). From computational simulations and experimental studies, it was hypothesized that the block threshold (i.e., minimum HFAC amplitude required) increases monotonically with frequency for all nerve fibers, and that small-diameter axons would have a higher block threshold than larger-diameter axons. These observations suggested that it would be impossible to selectively inhibit small-diameter axons with HFAC, as it would primarily block the large-diameter axon first. However, a more recent experiment on unmyelinated *Aplysia* axons (Joseph and Butera, 2009) showed that, once the HFAC frequency was higher than 12 kHz, the block threshold of these unmyelinated small-diameter axons would begin to decrease, instead of constantly increasing as previously believed. Therefore, findings from this *Aplysia* experiment provided a theoretical basis for selective inhibition of different axon types with different frequencies. In other words, with high enough HFAC frequency, the block threshold for small-diameter axons could be decreased to be lower than the threshold for large-diameter axons, which would permit selective inhibition of small-diameter axons. This hypothesis for selective inhibition was successfully verified in frogs and rats (Joseph and Butera, 2011; Patel and Butera, 2015). Additionally, researchers used *Aplysia* to demonstrate the possibility of combining HFAC with infrared neural inhibition (INI) to block the onset response during HFAC application (Lothet et al., 2014).

Second, neural tissue harvested from *Aplysia* can be kept viable with active neural signaling for many hours. The reason is that *Aplysia* lives in the intertidal zone. Thus, unlike many marine animals that live in the ocean, *Aplysia* are regularly found in tide pools that are exposed to significant changes in temperature, salinity (e.g., high salinity as pools dry out and low salinity during rain), and tidal surge. Since the animals do not maintain a fixed body temperature, and their soft bodies readily change volume in response to osmotic changes, their nervous systems, which are exposed to the animal’s open circulatory system, are very robust. Excised musculature and nervous systems from *Aplysia* can be maintained without anesthesia at room temperature. The excised tissue respires slowly, so it can be studied for many hours without significant changes in function. Although rarely mentioned in research articles, this advantage greatly extends the experimental time and increases the ability to collect longitudinal data from the same animal, and limits data noise due to inter-animal variance. These advantages are especially important for novel neuromodulation technologies, as researchers need to explore the parameter space to identify an effective configuration. As an example, it has been demonstrated that focused ultrasound (FUS) applied to the central nervous system can stimulate or inhibit neural activity in a wide range of vertebrate animal models, but the optimal paradigm for each type of effect is still yet to be determined due to the complexity of neural circuits in the brain (Baek et al., 2017; di Biase et al., 2019). In contrast, *Aplysia* ganglia consist of much smaller numbers of neurons and therefore provide a more tractable animal model for researchers to study. It has been reported that FUS can alter the excitation level of *Aplysia* neurons (Mori et al., 1987). In recent studies, an *ex vivo* FUS testing system used an isolated *Aplysia* ganglion and connected nerve, enabling the researchers to thoroughly explore the FUS parameter space on the same ganglion, and investigate the mechanisms of action (Jordan et al., 2022).

Third, in large *Aplysia*, the nerves between the ganglia may be many centimeters in length. The nerve length makes it possible to easily discriminate large-diameter axons from small-diameter axons based on the different conduction velocities. When stimulating a nerve, a compound action potential (CAP) can be evoked, which is the summation of action potentials conducted on all the different axons. Since axonal conduction velocity varies proportionally to the square root of axon diameter in unmyelinated axons, by the time the CAP reaches the other end of the long nerve, it naturally separates into different components. Researchers can explore the size selectivity of a given neuromodulation modality based on the response from different CAP components.

In addition, *Aplysia* neurons are large and can be repeatedly identified, which has been used to develop novel single-cell neural interfaces. *Aplysia* neurons have been used to develop multielectrode arrays (Regehr et al., 1989; Eggers et al., 1998), diamond electrodes (Halpern et al., 2006) and carbon fiber microelectrodes (Huan et al., 2021). In all of these studies, *Aplysia* was selected as the animal model because there are neural circuits that are thoroughly studied with standard electrophysiological methodologies that can be used as the gold standard for comparison. The surgical procedures are also simpler than in rats, mice, or other vertebrate animals, so that iterations of the novel neural interface design can be tested rapidly to quickly achieve the goal. Table 1 provides a comprehensive overview of the published literature that used *Aplysia* as a model system for the development of neuromodulation and related technologies.

**TABLE 1:** Summary of examples using *Aplysia* in the development of neurotechnologies.

Neurotechnology	Tissue type	Simplified summary	References
High-frequency alternating current (HFAC)	*Aplysia* pleural-abdominal connective	The threshold for inhibiting unmyelinated axons showed a unique decreasing trend when HFAC frequency was higher than 12 kHz	Joseph and Butera, 2009
HFAC combined with infrared neural inhibition (INI)	*Aplysia* pleural-abdominal connective nerve	HFAC combined with INI can produce onset-free neural inhibition	Lothet et al., 2014
Focused ultrasound (FUS)	*Aplysia* cerebral ganglia	FUS can alter the excitation level of *Aplysia* neurons	Mori et al., 1987
FUS	Isolated *Aplysia* abdominal ganglia and connected nerve	An *ex vivo* FUS testing system was built for exploring the FUS parameter space on isolated *Aplysia* ganglion and connected nerve	Jordan et al., 2022
Infrared neural inhibition (INI)	*Aplysia* buccal nerve and rat sciatic nerve	Parameters for spatially selective infrared neural inhibition were determined in *Aplysia* and were extended to rat sciatic nerve	Duke et al., 2013
INI	*Aplysia* buccal ganglia, pleural-abdominal connective and vagus nerve from musk shrew	Size-selective infrared neural inhibition protocols were developed in *Aplysia* and applied to musk shrew vagus nerve	Lothet et al., 2017
INI	*Aplysia* pleural-abdominal connective	Experiments confirmed that the theoretically predicted role of voltage-gated potassium ion channels was critical for INI	Ganguly et al., 2019a
INI	*Aplysia* pleural-abdominal connective	Experiments explored the optimal IR illumination length for minimizing the IR power threshold for INI	Ford et al., 2020, 2021
INI	*Aplysia* pleural-abdominal connective	Isotonic ion replacement can reduce the dose threshold of INI	Zhuo et al., 2021
Resistive heating and INI	*Aplysia* pleural-abdominal connective	Resistive heating can reproduce the selective infrared neural inhibition of small-diameter axons	Zhuo et al., 2022
Infrared neural stimulation (INS)	*Aplysia* buccal nerve and rat sciatic nerve	Demonstration of spatial and temporal selectivity of INS	Duke et al., 2012
Optical neural stimulation	*Aplysia* neuron and stained *Sepia* giant axon	Optical radiation with both visible (400–700 nm) and infrared (750–4,000 nm) light showed both excitatory and inhibitory effects	Arvanitaki and Chalazonitis, 1961
Optical neural stimulation	*Aplysia* abdominal ganglia	Laser radiation at 488 nm selectively stimulates neurons in the abdominal ganglion	Fork, 1971
Optical monitoring of neural activity	*Aplysia-*nerve trunks, abdominal ganglia, and eyes	Two membrane-associated dyes (WW375 and NK2367) were used to record neural activities by monitoring the change of their light absorption coefficient	Woolum and Strumwasser, 1978
Optical neural recording with fluorescent dyes	*Aplysia* buccal ganglia	Neuronal patterns of activity in the buccal ganglion of *Aplysia* were mapped by fluorescence imaging	Morton et al., 1991; Nakashima et al., 1992
Optical neural recording with fluorescent dyes	Cultured left upper quadrant neurons from the *Aplysia* abdominal ganglion	Experimental results demonstrated that prolonged asynchronous activity from synaptically interacting *Aplysia* neurons in culture can be recorded by fluorescence imaging	Parsons et al., 1989
Confocal fluorescence microscopy	*Aplysia* sensory neurons either in culture or in intact cell clusters	Experimental results demonstrated the value of fluorescence imaging of live cells for the spatial and temporal determination of the concentrations of second messengers.	Bacskai et al., 1993
Functional optical coherence tomography (fOCT)	*Aplysia* abdominal ganglia and nerves	The fOCT image provided significant scattering signal that correlates with neural activity	Lazebnik et al., 2003; Graf et al., 2009
Planar Microelectrode Array	*Aplysia* abdominal ganglia	A microelectrode array was built for extracellular recording from an *Aplysia* ganglion and spatial localization of identified cells in the ganglion	Novak and Wheeler, 1986
Multi-electrode array (MEA)	Cultured *Aplysia* neurons	A multi-electrode array was built to record and stimulate neural activity extracellularly	Regehr et al., 1989; Eggers et al., 1998
Gold-spine microelectrode array	Cultured left upper quadrant neurons from the *Aplysia* abdominal ganglion and cultured neurons from *Aplysia* buccal ganglion	A spine-shaped gold protrusion was developed to improve adhesion between electrode and neuron and achieve “intracellular-like” field potential recordings	Hai et al., 2009, 2010
Flexible microelectrode array	*Aplysia* buccal ganglia	A flexible microelectrode array was constructed to record action potentials and single- and multi-unit neural activities from the ganglionic surface	Sperry et al., 2018
Diamond electrode	*Aplysia* buccal ganglia and nerve	Diamond electrodes were developed for extracellular recording and stimulation	Halpern et al., 2006, 2010
Carbon fiber microelectrodes	*Aplysia* buccal ganglia and nerve	A carbon fiber microelectrode array demonstrated that the fibers could both provide stable multi-channel recording while stimulating and recording intracellularly	Huan et al., 2021
Implantable neural recording hardware	*Aplysia* buccal ganglia	A microcontroller-based wireless recording unit was developed for the recording of neural activity in seawater	Chestek et al., 2006
Pulse code modulation (PCM) microfluidic chips	*Aplysia* buccal and cerebral ganglia	Experiments demonstrated that PCM microfluidic chip could be used to apply chemical neural stimulation and induce rhythmic activity through the sheath of the ganglion	Azizi et al., 2010
Microscopic magnetic stimulation	*Aplysia* buccal ganglia and nerve	Microscopic magnetic stimulation demonstrated the capability to reversibly block action potentials in unmyelinated axons using a submillimeter magnetic coil. Modeling results showed that the microscopic magnetic stimulation caused a local depolarization that altered activation dynamics of the sodium channels and blocked neural conduction.	Skach et al., 2020
Magnetic resonance electrical impedance tomography (MREIT)	*Aplysia* abdominal ganglia	MREIT detected significant changes in ganglion images *in vitro* and could be used as a new modality to directly detect neural activity	Sadleir et al., 2019

## A detailed example: Exploring infrared neural inhibition for heat-based selective inhibition of small-diameter axons with *Aplysia*

A compelling example of how novel neural technologies can be effectively explored using *Aplysia* has been the recent exploration of modalities for selectively and reversibly inhibiting small-diameter axons using heat. The motivation for selective inhibition of small-diameter axons is the critical sensory role that small-diameter axons play in the mammalian nervous system. For example, nociceptive signals are carried by small-diameter unmyelinated C fibers (Besson and Chaouch, 1987), and the maintenance of homeostasis relies on controlling peripheral glands *via* small-diameter unmyelinated motor axons (Gabella, 1976). The dysfunction of small-diameter axons is related to neuropathic pain (Nickel et al., 2012), autonomic nervous system disorders (Phillips, 2005; Kishi, 2012), and other neural pathologies. Each of these small-fiber diseases adversely affects patients’ quality of life and work productivity, eventually putting a toll on the whole of society. Hence, the capability to selectively inhibit small-diameter axons without compromising the function of large-diameter axons is a critical unmet medical need.

**FIGURE 1:**
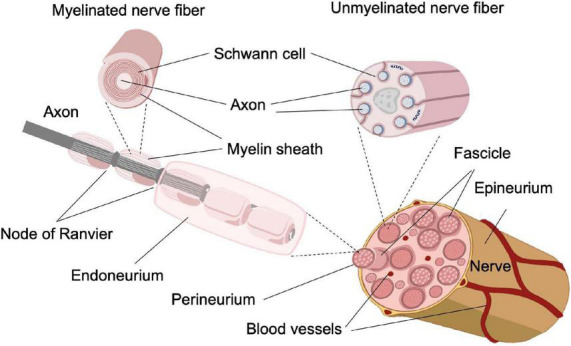
Schematic of the structure of a peripheral nerve (Guedan-Duran et al., 2020. The original work is published with permission under a Creative Commons Attribution 4.0 International License. To view a copy of this license, visit http://creativecommons.org/licenses/by/4.0/).

Selectively inhibiting small-diameter axons in vertebrate nerves is challenging because both small- and large-diameter axons are co-located in peripheral nerve fascicles, as shown in Figure 1. Consequently, inhibitory modalities that do not have size selectivity will block neural conduction of both small- and large-diameter axons. As the large-diameter axons are commonly associated with motor functions, a universal neural block could cause impairment of motor function and other unwanted side effects. Conventional pharmaceuticals for pain management not only lack selectivity for small-diameter axons, owing to their systemic distribution, but also lead to severe side effects, including addiction and overdosing that has led to the current opioid epidemic in the United States (Chou et al., 2015; Morrone et al., 2017; Vadivelu et al., 2018). Electrode-based neural modulation has been explored for selective inhibition of small-diameter axons. However, as the transmembrane potential evoked by extracellular electrodes is proportional to axon diameter (Rattay, 1986; Tai et al., 2009), it is an intrinsic property of electrode-based neuromodulation to affect the large-diameter axons first. Additional efforts such as multi-electrode arrays (Rozman et al., 1993; Lertmanorat and Durand, 2004) or changing the frequency of high-frequency alternating current (Joseph and Butera, 2011; Patel and Butera, 2015) are required for electrode-based neuromodulation to achieve selectivity on small-diameter axons, which increases the system’s complexity and hinders development. Therefore, a novel neural inhibitory modality that can selectively inhibit small-diameter axons while maintaining the functionality of large-diameter axons is required.

**FIGURE 2:**
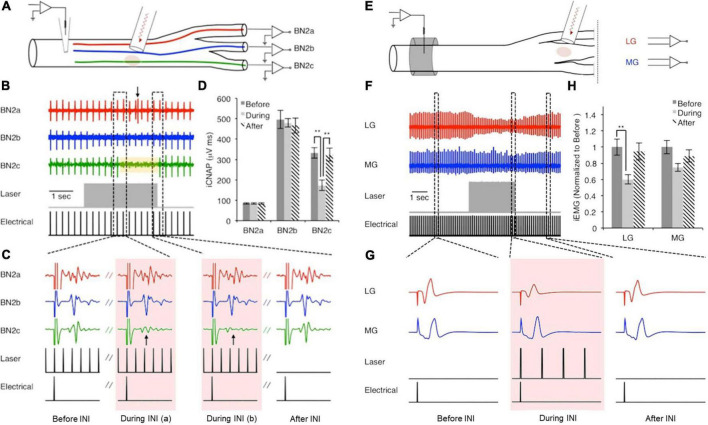
Nerve conduction block in buccal nerve 2 (BN2) of *Aplysia* **(A–D)** and in rat sciatic nerve **(E–H)**. Panels **(A–D)**: A train of low radiant exposure (0.50 ± 0.02 J/cm^2^), high frequency (200 Hz) infrared pulses (λ = 1,450 nm, pulse width = 0.2 ms) produced a rise in local tissue temperature and blocked responses projecting to BN2c. Panel **(A)**: Experimental setup; panel **(B)**: Overall view of the recorded responses; panel **(C)**: Representative signals showing the response before, during (arrow indicates block of CAP), and after the presence of infrared neural inhibition effect; panel **(D)**: Calculated area under the curve showed the spatial selective inhibitory effect. Panels **(E–H)**: Similarly, applying infrared pulses (same temporal parameters, 75.7 ± 5.3 mJ/cm^2^) to the tibial branch of the rat sciatic nerve, approximately 1 cm distal to the site of electrical stimulation, reduced evoked EMG amplitude of the lateral gastrocnemius (LG) but not the medial gastrocnemius (MG). Panel **(E)**: Experimental setup; panel **(F)**: Overall view of the recorded responses; panel **(G)**: Representative signals showing the response before, during, and after the infrared light application; panel **(H)**: Calculated area under the curve showed the spatial selective inhibitory effect. [Reproduced with permission from Duke et al. (2013). The original work is published under a Creative Commons Attribution-Non-commercial-NoDerivs 3.0 Unported License. To view a copy of this license, visit http://creativecommons.org/licenses/by-nc-nd/3.0/].

In 2012, Duke et al. (2012) first reported the inhibitory effect of pulsed infrared light application in *Aplysia* nerves, which they discovered while exploring temporal factors affecting the threshold of hybrid electro-optical stimulation. The inhibitory effect was further explored in *Aplysia*, since the anatomy of a buccal nerve and its branches make it tractable for testing the spatial selectivity of infrared neural inhibition (INI), as shown in Figure 2 (Duke et al., 2013). They demonstrated that infrared (IR) laser light could transiently and reversibly induce inhibition in *Aplysia* axons with precise spatial selectivity. As illustrated in Figure 2, electrical stimulation was applied to the proximal buccal nerve trunk and recording was done at all three distal branches. When the infrared laser was applied concurrently with electrical stimulation to one region of the proximal nerve trunk, neural conduction to the corresponding branch was blocked. Once the INI protocol was explored on *Aplysia* axons (Figures 2A–D), the investigators were able to use a nearly identical stimulation protocol to block action potential propagation on the tibial branch of the rat sciatic nerve (Figures 2E–H). The measured threshold temperature elevation for the inhibitory effect on both overall nerve compound action potential and muscle contraction was ∼7.0°C on *Aplysia* buccal nerve and ∼5.2°C on the tibial branch of rat sciatic nerve. When the infrared laser was turned off, the temperature rapidly dropped, and the inhibitory effect ceased. According to the temporal response test by Lothet et al., the inhibitory effect of INI is caused by a baseline temperature elevation (Lothet et al., 2017) rather than a spatiotemporal temperature gradient, which is critical for infrared neural stimulation (Wells J. et al., 2005). The robustness of the *Aplysia* nerve preparation permitted Duke et al. to develop their sophisticated experimental design consisting of four electrodes and two optical fibers, which they used to establish their infrared inhibition protocol and demonstrate its capabilities. As the authors wrote in their paper (Duke et al., 2013), “hours of intermittent stimulation” was done and the response was stable without visibly identifiable damage or significant change in the physiological recordings. This provided an ideal testing platform for the researchers to explore novel neuromodulation modalities without preparation run-down.

In 2017, Lothet et al. presented a general theory for how the effects of any modality that primarily acted on the axonal surface to modulate neural conduction could scale with axon diameter and induce size selectivity (Lothet et al., 2017, see the supplemental material). When a neuromodulation modality (e.g., INI), primarily affects the ion channels in the axon membrane, it was shown that the minimum required exposure length is proportional to the square root of the axon diameter. The theory therefore suggested that small-diameter axons would be more susceptible to inhibition induced by baseline temperature elevation than large-diameter axons. Ganguly and colleagues modeled and then experimentally demonstrated that the thermal acceleration of voltage-gated potassium ion channels was the critical factor during INI (Ganguly et al., 2019a,b). The mechanism of infrared inhibition is distinct from that of infrared stimulation, which depends on the rapid generation of a spatial and temporal temperature gradient induced by infrared neural stimulation (INS) that can cause changes in the membrane capacitance and evoke action potentials (Shapiro et al., 1980; Wells J. D. et al., 2005; Duke et al., 2012; Plaksin et al., 2018).

**FIGURE 3:**
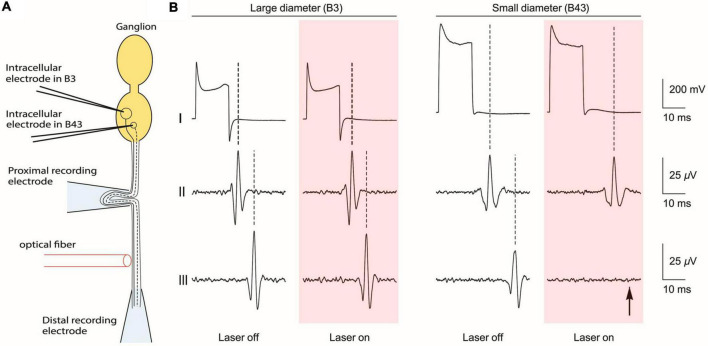
Direct demonstration of size-selective neural inhibition with B3 and B43 neurons in the *Aplysia* buccal ganglion. **(A)** Schematic of the experiment, which is aligned to the traces in part b of the figure. **(B)** Recordings from the soma (top), proximal recording electrode (middle) and the distal recording electrode (bottom). Pink boxes indicate the time of application of laser light. Intracellular electrical stimulation was applied to a large-diameter neuron (B3) and to a small-diameter neuron (B43), both of which have axons that project through a common nerve. When infrared laser light was applied *via* an optical fiber that was positioned between two extracellular recording electrodes on the nerve, only the neural conduction on the small-diameter axon was blocked (indicated by arrow). [Reproduced with permission from Lothet et al. (2017). The original work is published under a Creative Commons Attribution 4.0 International License. To view a copy of this license, visit http://creativecommons.org/licenses/by/4.0/].

To test the size selectivity of INI, Lothet et al. directly used individually identified neurons (B3 and B43) in *Aplysia* that had large and small diameter axons, respectively. As Figure 3 shows, the B3 and B43 neurons were electrically stimulated intracellularly while extracellular electrodes recorded propagation of their action potentials through a nerve both before and after an infrared laser application. When infrared light was delivered, only the small diameter axon was inhibited. The experiment was enabled by three unique features of *Aplysia* neurons. First, the average neuron soma size is larger than commonly studied mammalian neurons, making it easier to conduct intracellular stimulation. Second, there are a variety of neuron sizes accessible in the same ganglion, enabling the comparison between large and small-diameter axons. Third, those specific neurons with different sizes (B3 and B43) are identified and can be found across different individuals, making it possible to repeat the experiment with minimal variance. These features enabled the researchers to directly demonstrate the size-selectivity on small-diameter axons by infrared neural inhibition without ambiguity.

**FIGURE 4:**
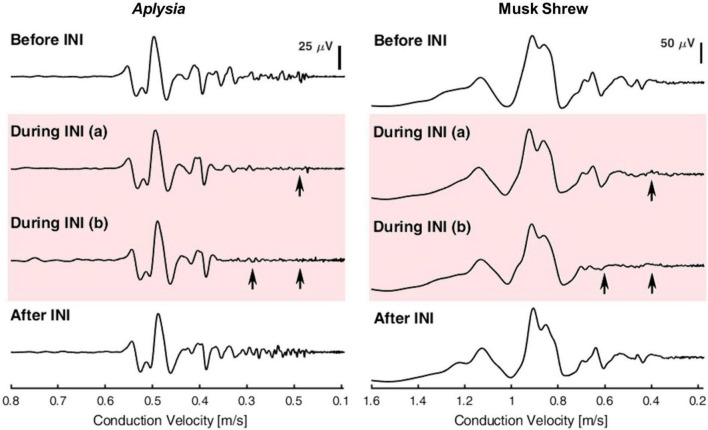
Demonstration of selective inhibition on small-diameter axons in *Aplysia* **(left)** and musk shrew **(right)**. Compound action potentials (CAPs) were evoked and recorded before, during, and after infrared neural inhibition (INI). Arrows indicate the selective inhibition effect on small-diameter axons that have a slower conduction velocity. The CAPs before and after INI remained similar, suggesting that the selective inhibition effect was reversible and the nerve’s health was not compromised acutely. Reproduced with permission from Lothet et al. (2017). The original work is published under a Creative Commons Attribution 4.0 International License. To view a copy of this license, visit http://creativecommons.org/licenses/by/4.0/].

Furthermore, after establishing size-selectivity during INI using single neuron stimulation, the size-selectivity of INI was verified at the whole nerve level using compound action potentials (CAPs). When stimulating the whole nerve, action potentials from all axons with different diameters can be evoked simultaneously and propagate along the axons throughout the length of the nerve. The summation of those action potentials recorded extracellularly forms the CAP. In unmyelinated axons, conduction velocity is proportional to the square root of the axon diameter (Jack et al., 1975). Therefore, as the CAP travels along the nerve, the latencies of different CAP components represent the response from different axon-size subpopulations. The long nerves of *Aplysia* combined with the relatively slow conduction velocity of unmyelinated axons permits researchers to easily differentiate the response from different axon-size subpopulations. Lothet et al. developed a size-selective infrared neural inhibition protocol for small-diameter axons in *Aplysia* that was successfully transferred to the vagus nerve in the musk shrew (*Suncus murinus*) (Figure 4). In contrast to *Aplysia*, individual nerve fibers had to be teased out of the vagus nerve to establish that they were unmyelinated C fibers (Lothet et al., 2017). It is also worth noting that owing to species difference, the baseline temperature elevation threshold for size-selective inhibition on small-diameter axons was reduced from ∼9°C to ∼3°C after transferring the INI protocol from *Aplysia* nerve to the vagus nerve of musk shrew (Lothet et al., 2017).

**FIGURE 5:**
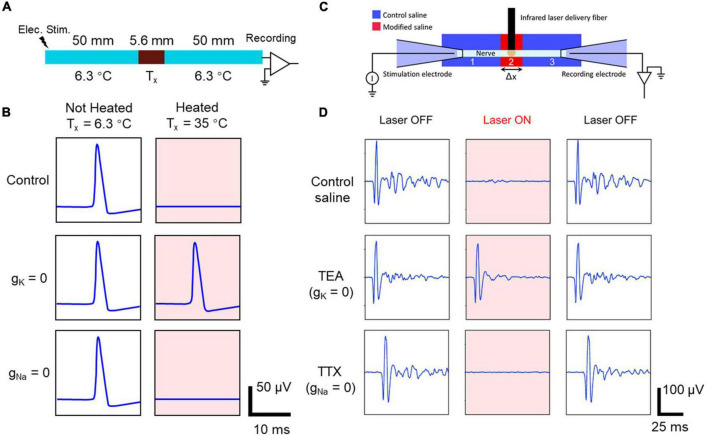
Computational modeling **(A,B)**, and experimental validation in an *Aplysia* nerve **(C,D)** of the effect of blocking different ion channels on the heat-induced neural block. Panel **(A)**: A schematic of a model axon with a central region where the temperature can be locally elevated (representing the thermal effect during IR application) and where the ion channel conductance can be set to zero (representing the local application of an ion channel blocker). Panel **(B)**: The modeled action potential recordings under different conditions. Only when voltage-gated potassium channels are blocked (*g*_K_ = 0) is the heat-induced block effect reversed, allowing the action potential to propagate through the heated area and be recorded at the distal end of the axon (right hand pink panels), suggesting that the heat-induced block requires voltage-gated potassium channels. Panel **(C)**: An experimental schematic of an *ex vivo* electrophysiology test using an *Aplysia* nerve. IR light was applied to the central region of the nerve, which was also exposed to modified saline with different ion channel blockers. Panel **(D)**: The recorded CAPs during different combinations of IR light application and ion channel blockers. In agreement with the modeling prediction, only when TEA was applied to block the voltage-gated potassium ion channels was the IR neural inhibition effect reversed, allowing the action potential to propagate through the heated area and be recorded at the distal end of the axon. Reproduced from Ganguly et al., 2019a,b with permission. The original works are licensed under the terms of the Creative Commons Attribution 3.0 License and Creative Commons Attribution 4.0 Unported License, respectively. See license detail at https://creativecommons.org/licenses/by/3.0/ and https://creativecommons.org/licenses/by/4.0/].

Ganguly et al. (2019a) used a NEURON simulation of a squid giant axon to demonstrate that a likely mechanism for thermal inhibition was the acceleration of the kinetics of the voltage-gated potassium ion channels, resulting in a rapid depolarization-activated hyperpolarization due to elevated temperature. They showed that this inhibitory effect would be greater in smaller-diameter axons (see Figures 5A,B).

Ganguly et al. (2019b) then provided experimental evidence for the hypothesis using *Aplysia* nerves (see Figures 5C, D). As the *Aplysia* axon and squid giant axon are both unmyelinated axons with different diameters, the qualitative conclusions from the modeling work should apply directly to *Aplysia* axons. As Figure 5D shows, selectively blocking voltage-gated potassium ion channels (using TEA) eliminated the thermal inhibition, whereas blocking voltage-dependent sodium ion channels (using TTX) did not prevent thermal inhibition. Thus, experimental evidence from *Aplysia* confirmed the modeling work based on the squid giant axon and demonstrated that the infrared neural inhibition relies on the accelerated potassium-channel kinetics during baseline temperature elevation induced by the absorption of infrared light by the nerve. The success of the experiment relies on *Aplysia*’s unmyelinated axons of different diameters, providing an animal model in which the modeling conclusions from a Hodgkin-Huxley model and its variants can be directly tested in the laboratory.

These studies were extended by Ford et al. (2020, 2021) showing that one could reduce the IR dose required for thermal inhibition by illuminating a greater length of the nerve, and by Zhuo et al. (2021) showing that ion substitution could also reduce the dose of laser light needed for thermal inhibition. Both studies explored the parameter space to determine the change in IR threshold, which required a substantial number of repeated experiments on the same nerve to avoid variation between animals. The robustness of the *Aplysia* preparation enabled the two studies to be conducted in a reasonable amount of time and with a minimal number of animals. Most recently, Zhuo et al. (2022) have demonstrated that selective IR inhibition can be reproduced by resistive heating. The *ex vivo Aplysia* model provided a robust testing platform for the researchers to apply both heating modalities with different levels of power on the same nerve sequentially and compare the responses, which helped minimize the impact of variance between the animals and lowered the number of animals needed.

## Differences between *Aplysia* and vertebrate neurons

Given all these advantages, *Aplysia* should be considered a useful model system for developing novel neuromodulation technologies. It is also important to consider the similarities and differences between *Aplysia*, other model animals, and humans. Two major differences are (1) *Aplysia* axons lack myelination and (2) *Aplysia’s* nervous system consists of several separated ganglia rather than a single complex brain connected to a peripheral nervous system. It is worth noting, however, that the peripheral ganglia in vertebrates and humans have some similarities to those in *Aplysia*. The unmyelinated *Aplysia* axon can serve as a good model of the small-diameter unmyelinated C fibers in vertebrates. The left and right pleural-abdominal connectives in *Aplysia* consist of 1,388 and 1,832 axons, respectively, with similar axon diameter distribution ranging from 0.1 to > 25 μm with the majority of axons less than 4 μm in diameter (Frazier et al., 1967; Bedini and Geppetti, 2000). The range of *Aplysia* axon diameters covers the entire diameter range of axons in vertebrates, while the majority of axon diameters are similar to the diameters of unmyelinated C fibers (Terzis et al., 1991), which range from 0.5 to 2 μm. Also, the lack of myelination limits the conduction velocity of *Aplysia* axons to about 0.1–0.7 m/s (Lothet et al., 2017), which is similar to the conduction velocities of unmyelinated C fibers in vertebrates (Terzis et al., 1991). Previous studies have demonstrated that infrared neural inhibition can be developed using *Aplysia* and then migrated to vertebrate animals (Duke et al., 2013; Lothet et al., 2017) to selectively inhibit the slow-conducting axons, which are primarily the unmyelinated C fibers. The threshold for inhibition of myelinated larger-diameter axons is higher due to the presence of myelin, which blocks the penetration of infrared light and limits the interaction between infrared light and voltage-gated ion channels to the nodes of Ranvier. Similarly, the myelination of axons contributes to the limited sensitivity of vertebrate nerves to infrared neural stimulation (Duke et al., 2012; Peterson and Tyler, 2013).

*Aplysia* has the basic complement of ion channels found in neurons throughout evolution (Franciolini and Petris, 1989; Moran et al., 2015). There is a common ancestral four-domain voltage-gated cation channel (FVCC) that evolved into all eukaryotic FVCC subfamilies (Pozdnyakov et al., 2018). Different voltage-gated channels (Nav1, Nav2, Cav1, Cav2, and Cav3) and leak channels (NALCN) all share a common 24 transmembrane pore segment (4 × 6 TM) template and the change of ion-selectivity only requires a single lysine residue change in the ion selectivity filter domain (Fux et al., 2018). Research on *Aplysia’s* potassium channel also showed that potassium channels originated much earlier, before the split of mollusks and arthropods (Zhao, 1993). Like other invertebrates, *Aplysia* possesses both Nav1 and Nav2 ion channel families, whereas vertebrates only possess the Nav1 family (Zakon, 2012). The Nav1 family has expanded significantly during vertebrate evolution and gained the capability to cluster at axon initial segments and the nodes of Ranvier of myelinated axons (Zakon, 2012; Fux et al., 2018). The similarity of ion channel proteins would allow neural manipulation of many *Aplysia* ion channels to be robustly translatable to vertebrates and even humans. Moreover, if there are differences, this could highlight the unique aspects of vertebrate and human biophysics, such as the unique diversity of the Nav1 family in vertebrates.

The similarity and differences between *Aplysia* and vertebrates also exist in other aspects of their molecular biology. A study of the *Aplysia* transcriptome showed that the evolutionary distance from humans to *Aplysia* is shorter than the distance from humans to *Drosophila* and *C. elegans* (Moroz et al., 2006). Another recent comparative transcriptome study showed that *Aplysia* possesses synaptic proteins very similar to those of *Octopus*, whereas several synaptic scaffold protein families in both mollusks are missing in vertebrate lineages (Orvis et al., 2022). The similarity in synaptic proteins and difference in neural network structure (separated ganglia vs. an elaborated central brain) between *Aplysia* and *Octopus* suggested that the difference in cognitive capacity is more related to the difference in neural network structure than to the lower-level differences of molecular biophysics (Orvis et al., 2022). Another recent study mapped the *Aplysia* proteome to humans and cross-referenced it with two databases of genes of interest in Alzheimer’s disease research. The results identified 898 potential orthologs of interest in *Aplysia*, 59 of which showed concordant differential expression across species (Kron and Fieber, 2022). *Aplysia* provides an experimentally tractable animal platform for researchers to explore neuromodulation techniques that rely on basic physiological mechanisms. The differences in neural network structure and cognitive capability between *Aplysia* and vertebrates present both a challenge and a potential negative control for exploring the mechanism of diseases and neuromodulation techniques.

Thus, while *Aplysia* is a useful animal model that lends itself well to initial validation of neuromodulatory devices and optimization of parameters, in the translation of the findings from *Aplysia* studies, it is likely that investigators will have to adjust and modify the details of the approach and parameters in order to optimize these for the specific applications, anatomy, physiology, and geometry of their system of interest.

## Summary

Overall, these results demonstrate that *Aplysia californica* as a tractable experimental system has several unique advantages: Neurons that can be repeatedly identified and excited; neural tissues that can last for a long period of time in an *ex vivo* experimental setup; nerves consisting of pure unmyelinated axons with different diameters; and neural circuits that are well-studied and that permit different levels of experimental design (from single neuron tests to whole behavioral tests). Generally, *Aplysia* is suitable for testing modalities in which the basic biophysics of that modality is likely to be similar across species (e.g., responses to temperature or changes in ionic concentration), and during initial tests of novel modalities in which the exploration of parameter spaces and the determination of fundamental mechanisms are likely to be broadly applicable across species. As discussed in this review, previous studies have demonstrated that novel technologies that interface with and manipulate the nervous system can be developed using *Aplysia* and then migrated to vertebrate animals (e.g., Duke et al., 2013; Lothet et al., 2017). Overall, the use of invertebrate animal models, such as *Aplysia californica*, should be considered an important tool for the development of novel neural interfaces for neuromodulation.

## Author contributions

JZ and HJC wrote the review and prepared the figures. JG revised the review and figures. EJ, HJC, and MJ critically revised the manuscript. All authors contributed to the article and approved the submitted version.
